# Effects of post-deposition annealing ambient on band alignment of RF magnetron-sputtered Y_2_O_3_ film on gallium nitride

**DOI:** 10.1186/1556-276X-8-53

**Published:** 2013-01-29

**Authors:** Hock Jin Quah, Kuan Yew Cheong

**Affiliations:** 1 Energy Efficient & Sustainable Semiconductor Research Group, School of Materials and Mineral Resources Engineering, Universiti Sains Malaysia, Engineering Campus, Nibong Tebal, Seberang Perai Selatan, Penang, 14300, Malaysia

**Keywords:** Yttrium oxide, Gallium nitride, Post-deposition annealing, Band alignment, Conduction band offset

## Abstract

The effects of different post-deposition annealing ambients (oxygen, argon, forming gas (95% N_2_ + 5% H_2_), and nitrogen) on radio frequency magnetron-sputtered yttrium oxide (Y_2_O_3_) films on n-type gallium nitride (GaN) substrate were studied in this work. X-ray photoelectron spectroscopy was utilized to extract the bandgap of Y_2_O_3_ and interfacial layer as well as establishing the energy band alignment of Y_2_O_3_/interfacial layer/GaN structure. Three different structures of energy band alignment were obtained, and the change of band alignment influenced leakage current density-electrical breakdown field characteristics of the samples subjected to different post-deposition annealing ambients. Of these investigated samples, ability of the sample annealed in O_2_ ambient to withstand the highest electric breakdown field (approximately 6.6 MV/cm) at 10^−6^ A/cm^2^ was related to the largest conduction band offset of interfacial layer/GaN (3.77 eV) and barrier height (3.72 eV).

## Background

Increasing concerns regarding the escalating demand of energy consumption throughout the world has triggered the needs of developing energy-efficient high-power and high-temperature metal-oxide-semiconductor (MOS)-based devices. It has been projected that gallium nitride (GaN) has the potential of conforming to the needs of these MOS-based devices due to its promising properties, which include wide bandgap (3.4 eV), large critical electric field (3 MV/cm), high electron mobility, as well as good thermal conductivity and stability [1-6]. The fabrication of a functional GaN-based MOS device requires a high-quality gate oxide that is capable of resisting a high transverse electric field [7,8]. Native oxide (Ga_2_O_3_) of GaN [9-13] and a relatively low-dielectric-constant (*k*) SiN_*x*_O_*y*_[2] or SiO_2_[14-19] have been successfully grown and deposited, respectively, as gate oxides in GaN-based MOS devices. However, these gate oxides are not the preferred choices. The shortcoming encountered by the former gate is the slow growth gate, high oxidation temperature (>700°C), and high leakage current [12,13] while the latter gate with a relatively low *k* is unable to withstand the high electric field imposed on GaN [7,20,21]. Thereafter, numerous high-*k* gate oxides [3,20-28] have been selected for investigation on GaN-based MOS devices. Recent exploration on the employment of radio frequency (RF) magnetron-sputtered Y_2_O_3_ gate subjected to post-deposition annealing (PDA) from 200°C to 1,000°C for 30 min in argon ambient has revealed that the Y_2_O_3_ gate annealed at 400°C has yielded the best current density-breakdown field (*J-E*) characteristic as well as the lowest effective oxide charge, interface trap density, and total interface trap density [25]. It is noticed that the acquired *J-E* characteristic for this sample is better than majority of the investigated gate oxide materials [25]. The ability of Y_2_O_3_/GaN MOS structure to be driven at a high *E* and low *J* is attributed to the fascinating properties possessed by Y_2_O_3_, such as high *k* value (*k* = 12 to 18), large bandgap (approximately 5.5 eV), and large conduction band offset (approximately 1.97 eV) [25,29-31]. Despite that, the presence of oxygen-related defects, changes in compositional homogeneity of Y_2_O_3_, and formation of interfacial layer (IL) are of particular concern as either of these factors might alter the bandgap of Y_2_O_3_ and band alignment of Y_2_O_3_ with respect to the GaN, which would influence the *J-E* characteristic of the MOS structure. Li et al. has reported previously that *J-E* characteristic of the MOS structure is dependent on the thickness of IL, wherein interface quality of the atomic layer deposited HfO_2_ on Si can be altered via the IL thickness [32]. In order to reduce oxygen-related defects and restore compositional homogeneity of Y_2_O_3_, it is essential to perform post-deposition annealing on the oxide [33]. Besides, the oxygen content near the Y_2_O_3_/GaN interface can be regulated by varying the post-deposition annealing ambient and eventually controlling the formation of IL. Therefore, engineering of the bandgap of Y_2_O_3_ gate and band alignment of Y_2_O_3_ with GaN through different PDA ambients is of technological importance. In this work, effects of different PDA ambients (oxygen (O_2_), argon (Ar) [25], nitrogen (N_2_), and forming gas (FG; 95% N_2_ + 5% H_2_)) at 400°C for 30 min on the Y_2_O_3_/GaN structure in modifying the bandgap of Y_2_O_3_ gate and band alignment of Y_2_O_3_/GaN are presented. A correlation on the bandgap of Y_2_O_3_ gate and band alignment of Y_2_O_3_/GaN with regard to the *J-E* characteristics is also discussed in this paper.

## Methods

**Figure 1:**
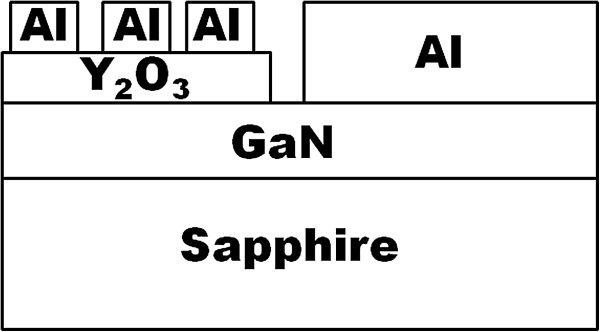
**Al/Y**_**2**_**O**_**3**_**/GaN MOS test structure.**

Prior to the deposition of 60-nm thick Y_2_O_3_ films on the commercially purchased Si-doped (n-type) GaN epitaxial layers with thickness of 7 μm and doping concentration of 1 to 9 × 10^18^ cm^−3^ grown on sapphire substrates, the wafer, which was diced into smaller pieces, were subjected to RCA cleaning. Subsequently, these samples were loaded into a vacuum chamber of RF magnetron sputtering system (Edwards A500, Edwards, Sanborn, NY, USA). A comprehensive description on the deposition process of Y_2_O_3_ films has been reported elsewhere [29,30]. Then, PDA was performed in a horizontal tube furnace at 400°C in different ambients (O_2_, Ar, N_2_, and FG (95%N_2_ + 5% H_2_)) for 30 min. The heating and cooling rate of approximately 10°C/min was used for the PDA process. After the PDA process, X-ray photoelectron spectroscopy (XPS) measurements were conducted on the samples at the Research Center for Surface and Materials Science, Auckland University, New Zealand, using Kratos Axis Ultra DLD (Shimadzu, Kyoto, Japan) equipped with a monochromatic Al-K_α_ X-ray source (*hv* = 1486.69 eV). The spectra of the survey scan were obtained at a low pass energy of 160 eV with an energy resolution of 0.1 eV, and the photoelectron take-off angle was fixed at 0° with respect to the surface normal. Chemical depth profiling was performed by etching the samples using an Ar ion gun operated at 5 keV in order to identify the boundary of Y_2_O_3_ and interfacial layer between the oxide and GaN. To further determine the bandgap of Y_2_O_3_ and IL, a detailed scan of O 1*s* was first performed at the same pass energy of 20 eV with an energy resolution of 1.0 eV. The energy loss spectrum of O 1*s* would provide the bandgap of Y_2_O_3_ and IL by taking into consideration the onset of a single particle excitation and band-to-band transition. Kraut’s method was utilized in the extraction of the valence band offset of Y_2_O_3_ and IL [34,35]. In order to fabricate MOS test structure, the Y_2_O_3_ film was selectively etched using HF/H_2_O (1:1) solution. Next, a blanket of aluminum was evaporated on the Y_2_O_3_ film using a thermal evaporator (AUTO 306, Edwards). Lastly, an array of Al gate electrode (area = 2.5 × 10^−3^ cm^2^) was defined using photolithography process. Figure 1 shows the fabricated Al/Y_2_O_3_/GaN-based MOS test structure. The current–voltage characteristics of the samples were measured using a computer-controlled semiconductor parameter analyzer (Agilent 4156C, Agilent Technologies, Santa Clara, CA, USA).

## Results and discussion

**Figure 2:**
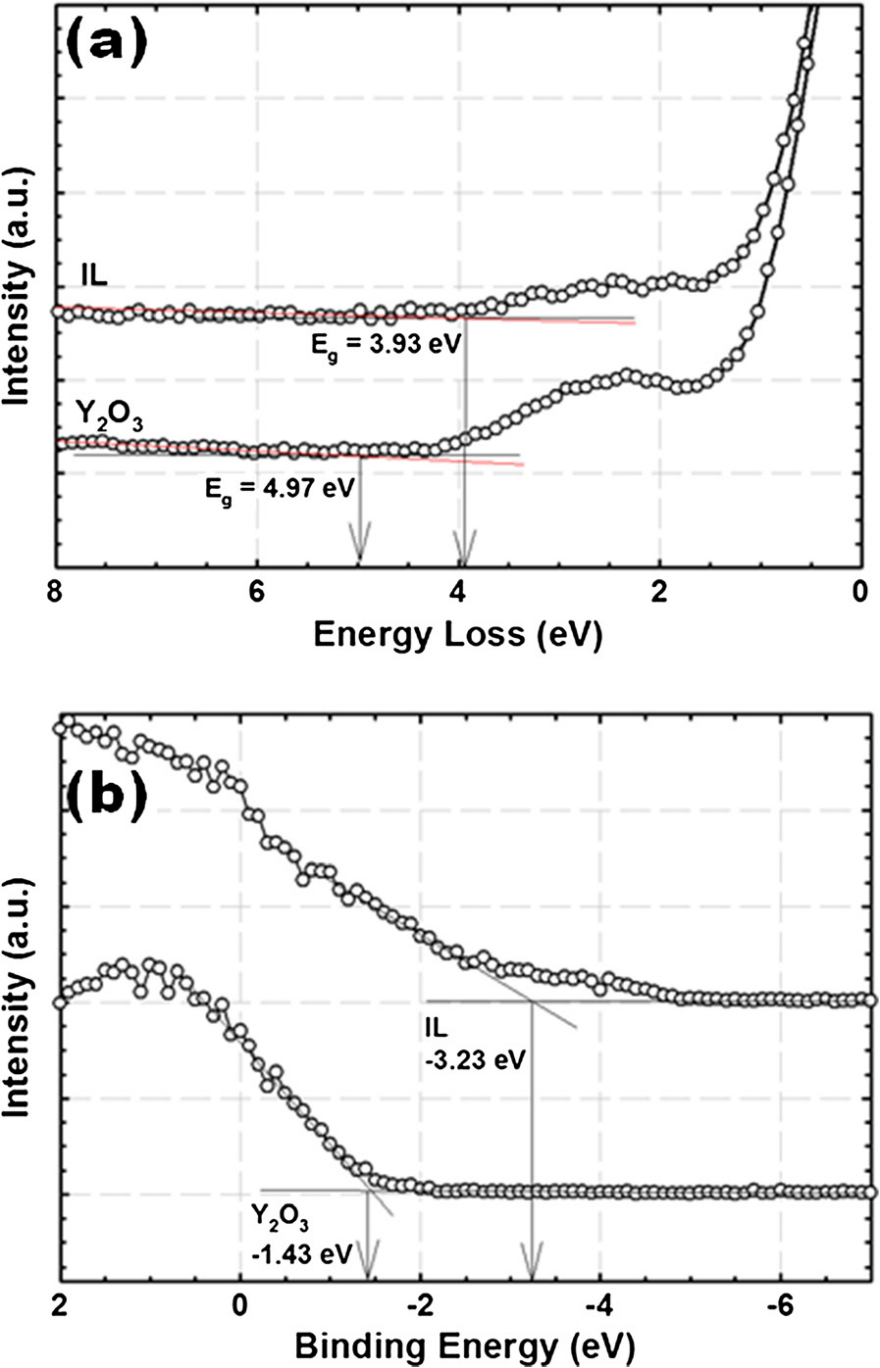
**XPS O 1*s *energy loss and valence band photoelectron spectrum.** (**a**) Typical XPS O 1*s* energy loss spectrum of Y_2_O_3_ and interfacial layer for the sample annealed in O_2_ ambient. (**b**) Typical valence band spectrum of Y_2_O_3_ and interfacial layer for the sample annealed in O_2_ ambient.

**Figure 3:**
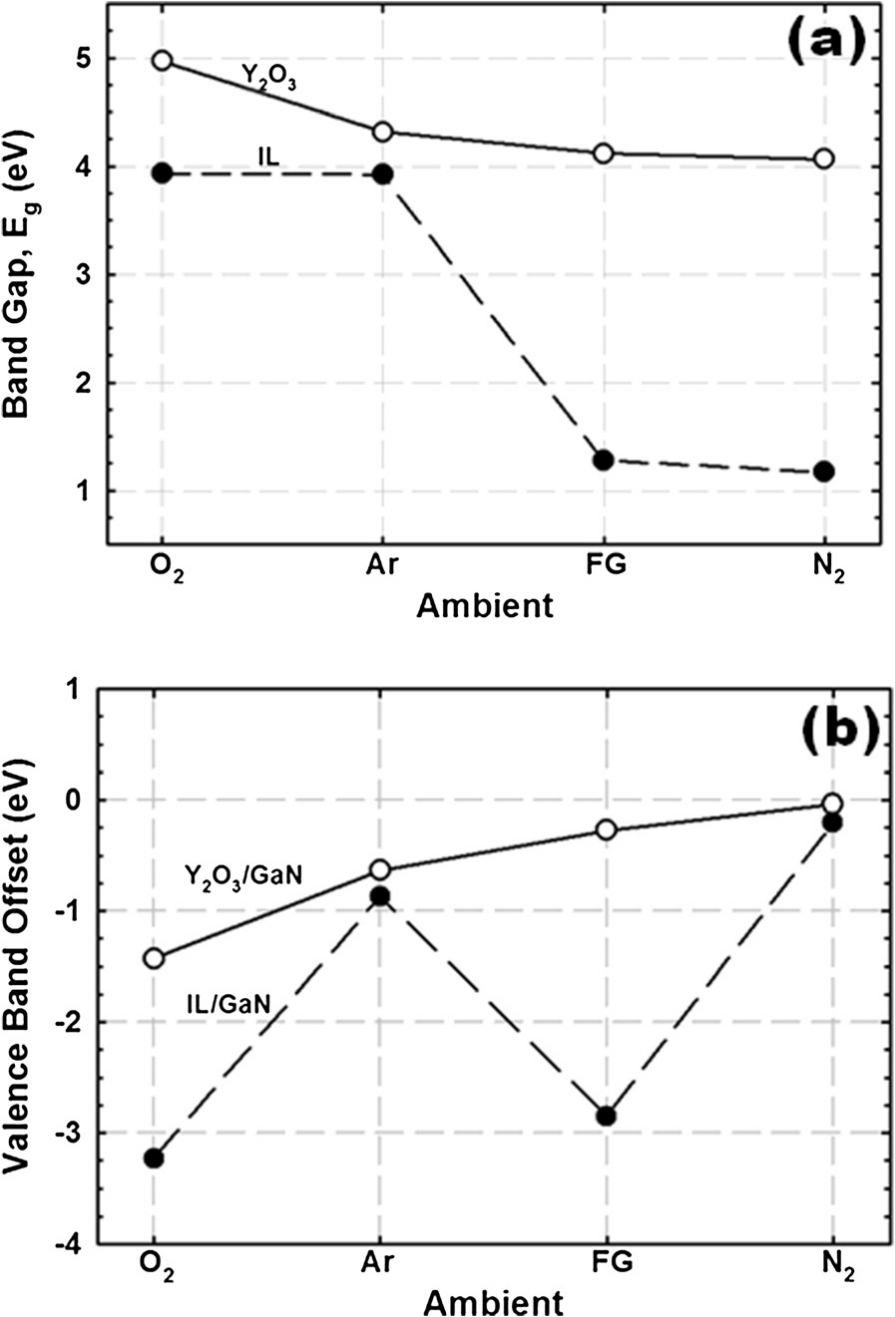
**Bandgap and valence band offset of Y**_**2**_**O**_**3 **_**and interfacial layer.** (**a**) Bandgap of Y_2_O_3_ and IL for the sample annealed in different ambients. (**b**) Valence band offset of Y_2_O_3_/GaN and IL/GaN as a function of post-deposition annealing ambient.

Bandgap (*E*_g_) values for Y_2_O_3_ and IL are extracted from the onset of the respective energy loss spectrum of O 1*s* core level peaks. The determination of *E*_g_ values for Y_2_O_3_ and IL is done using a linear extrapolation method, wherein the segment of maximum negative slope is extrapolated to the background level [36]. Figure 2a shows typical O 1*s* energy loss spectra of Y_2_O_3_ and IL for the sample annealed in O_2_ ambient. The extracted *E*_g_ values are in the range of 4.07 to 4.97 eV and 1.17 to 3.93 eV with a tolerance of 0.05 eV for Y_2_O_3_ and IL, respectively, for samples annealed in different post-deposition annealing ambients (Figure 3a).

Typical valence band photoelectron spectra of Y_2_O_3_ and IL for the sample annealed in O_2_ ambient are presented in Figure 2b. By means of linear extrapolation method, the valence band edges (*E*_v_) of Y_2_O_3_ and IL could be determined by extrapolating the maximum negative slope to the minimum horizontal baseline [36]. The acquired valence band offset (Δ*E*_v_) values of Y_2_O_3_ and IL with respect to GaN substrate are in the range of −0.04 to −1.43 eV and −0.21 to −3.23 eV with a tolerance of 0.05 eV, respectively, for all of the investigated samples. The Δ*E*_v_ values of Y_2_O_3_/GaN and IL/GaN are shown in Figure 3b as a function of PDA ambient.

**Figure 4:**
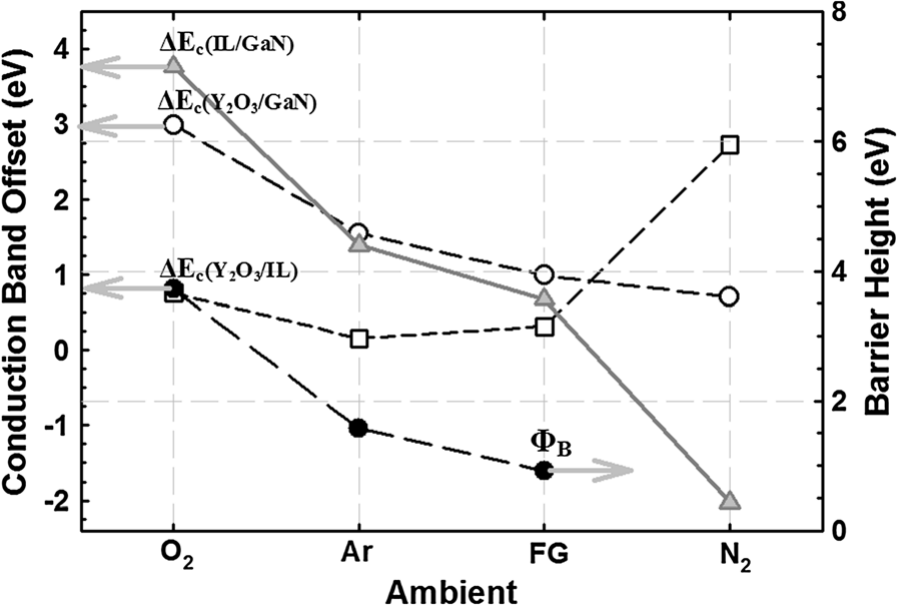
Conduction band offset and barrier height for samples annealed in different ambients.

The determination of both *E*_g_ of Y_2_O_3_ and IL as well as Δ*E*_v_ of Y_2_O_3_/GaN and IL/GaN enables the calculation of the conduction band offset (Δ*E*_c_) of Y_2_O_3_/GaN, IL/GaN, and Y_2_O_3_/IL using the following equation: Δ*E*_c_(oxide or IL) = *E*_g_(oxide or IL) − Δ*E*_v_(oxide/GaN or IL/GaN) − *E*_g_(GaN), where *E*_g(GaN)_ is 3.40 eV for GaN [37]. The obtained values of Δ*E*_c_(Y_2_O_3_/GaN), Δ*E*_c_(IL/GaN), and Δ*E*_c_(Y_2_O_3_/IL) for all of the investigated samples are presented in Figure 4. In general, a reduction in *E*_g_(Y_2_O_3_), *E*_g_(IL), Δ*E*_c_(Y_2_O_3_/GaN), and Δ*E*_c_(IL/GaN) is observed when different PDA ambients are performed, as indicated by O_2_ > Ar > FG > N_2_. The IL has been proven using XPS to be comprised of a mixture of Ga-O, Ga-O-N, Y-O, and Y-N bonding (HJQ and KYC, unpublished work). The detection of Ga-O and Ga-O-N bonding in the region of IL indicates that the oxygen dissociated from Y_2_O_3_ during PDA in different ambients would diffuse inward to react with the decomposed GaN substrate. During PDA in O_2_ ambient, an additional source of oxygen from the gas ambient has contributed to the formation of Ga-O and Ga-O-N bonding in the region of IL. Sample subjected to PDA in O_2_ ambient attains the largest *E*_g_(Y_2_O_3_) and *E*_g_(IL) as well as the highest values of Δ*E*_c_(Y_2_O_3_/GaN) and Δ*E*_c_(IL/GaN). This is related to the supply of O_2_ from the gas ambient during PDA, which has contributed to the reduction of oxygen-related defects in the Y_2_O_3_ film and the improvement in the compositional homogeneity of the oxide film. The absence of O_2_ supply during PDA in Ar (inert) and reducing ambient, such as FG and N_2_, may be the reason contributing to the attainment of lower *E*_g_(Y_2_O_3_), *E*_g_(IL), Δ*E*_c_(Y_2_O_3_/GaN), and Δ*E*_c_(IL/GaN) values than the sample annealed in O_2_. The presence of N_2_ in both FG and N_2_ ambient has caused the formation of O_2_-deficient Y_2_O_3_ film, wherein N atoms dissociated from N_2_ gas may couple with the oxygen-related defects in the Y_2_O_3_ film [30,38]. In addition, the presence of N_2_ in both FG and N_2_ ambient is also capable of performing nitridation process to diminish the tendency of O_2_ dissociated from the Y_2_O_3_ film during PDA to diffuse inward and react with the GaN substrate [30]. Thus, the interfacial layer formed in between the Y_2_O_3_/GaN structure for these samples could be O_2_ deficient. Despite the fact that FG and N_2_ ambient are capable of providing nitridation and coupling process, the percentage of N_2_ in FG ambient (95% N_2_) is lower than that in pure N_2_. Hence, PDA in N_2_ ambient will enhance the nitridation process and coupling of N atoms with the oxygen-related defects in Y_2_O_3_, which leads to the formation of more O_2_-deficient Y_2_O_3_ film and IL when compared with the sample annealed in FG ambient. This could be the reason leading to the attainment of the lowest *E*_g_(Y_2_O_3_), *E*_g_(IL), Δ*E*_c_(Y_2_O_3_/GaN), and Δ*E*_c_(IL/GaN) values for the sample annealed in N_2_ ambient.

**Figure 5:**
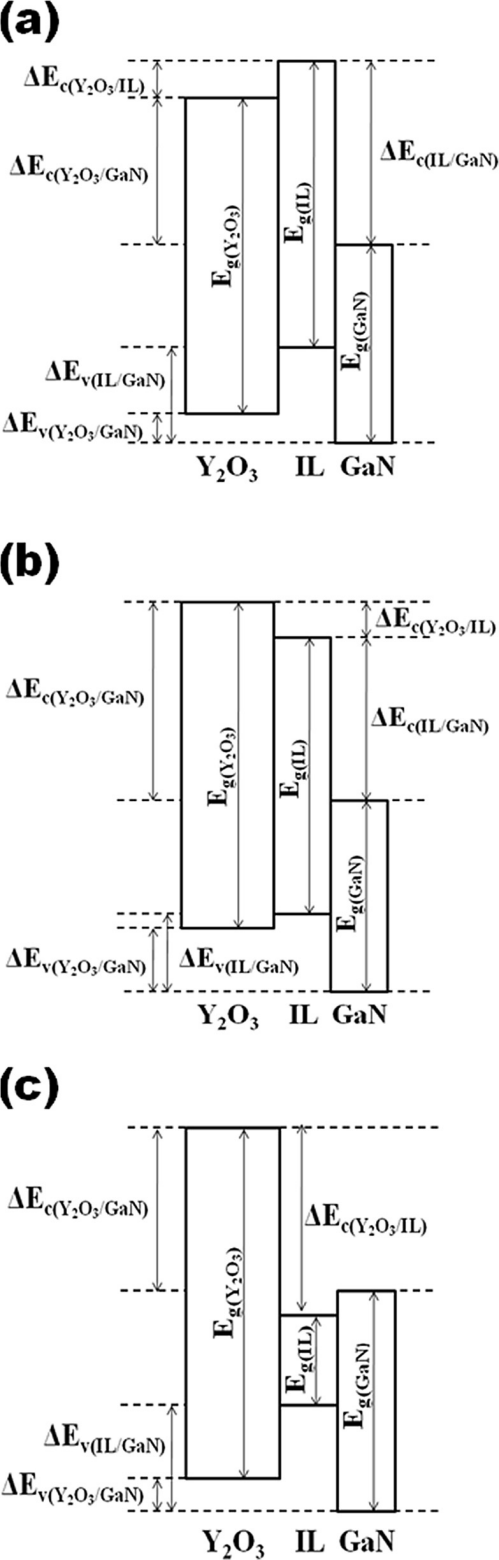
**Schematic diagram showing the energy band alignment of the Y**_**2**_**O**_**3**_**/IL/GaN system.** Energy band alignment of the Y_2_O_3_/IL/GaN system for the sample annealed in (**a**) oxygen, (**b**) argon and forming gas, and (**c**) nitrogen ambient.

**Figure 6:**
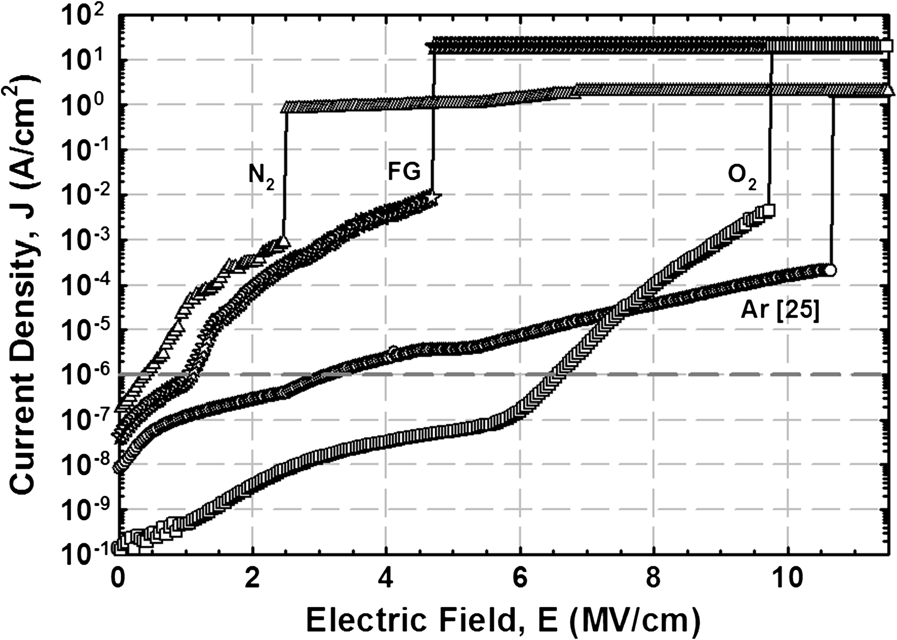
**Comparison of*J*-*E *characteristics of Al/Y**_**2**_**O**_**3**_**/IL/GaN-based MOS capacitors.**

A schematic illustration of the energy band alignment of the Y_2_O_3_/IL/GaN structure that had been subjected to different PDA ambients is presented in Figure 5. Three different energy band alignment structures were obtained due to the effect of PDA ambient. It is noticed that the conduction band edge of IL is higher than that of Y_2_O_3_ for the sample annealed in O_2_ ambient, but it is lower in samples annealed in Ar, FG, and N_2_ ambient. This band alignment shift would influence the leakage current density-electrical field (*J-E*) characteristics of the samples (Figure 6). The dielectric breakdown field (*E*_B_) is defined as the electric field that causes a leakage current density of 10^−6^ A/cm^2^, which is not related to a permanent oxide breakdown but representing a safe value for device operation [39]. Of all the investigated samples, the sample annealed in O_2_ ambient demonstrates the lowest *J* and the highest *E*_B_ (approximately 6.6 MV/cm) at *J* of 10^−6^ A/cm^2^. This might be attributed to the attainment of the largest *E*_g_(Y_2_O_3_) and *E*_g_(IL) as well as the highest values of Δ*E*_c_(Y_2_O_3_/GaN) and Δ*E*_c_(IL/GaN), while for other samples, a deterioration in *J* and *E*_B_ is perceived. The reduction is ranked as Ar > FG > N_2_.

**Figure 7:**
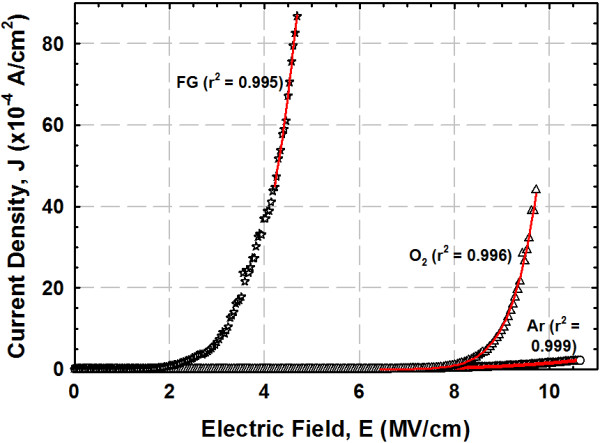
**Experimental data fitted well with FN tunneling model.** Experimental data (symbol) of samples annealed in O_2_, Ar (HJQ and KYC, unpublished work), and FG ambient fitted well with FN tunneling model (line).

In order to determine whether the *E*_B_ of the investigated samples is either dominated by the breakdown of IL, Y_2_O_3_, or a combination of both Y_2_O_3_ and IL, Fowler-Nordheim (FN) tunneling model is employed to the extract barrier height (Φ_B_) of Y_2_O_3_ on GaN. FN tunneling mechanism is defined as tunneling of the injected charged carrier into the conduction band of the Y_2_O_3_ gate oxide via passing through a triangular energy barrier [7,8,30]. This mechanism can be expressed as *J*_FN_ = *AE*^2^exp(−*B*/*E*), where *A* = *q*^3^*m*_o_/8(*hm*Φ_B_, *B* = 4(2 m)^1/2^ Φ_B_^3/2^/(3*qh*/2), *q* is the electronic charge, *m* is the effective electron mass in the Y_2_O_3_ (*m* = 0.1*m*_o_, where *m*_o_ is the free electron mass), and *h* is Planck’s constant [8,40]. In order to fit the obtained experimental data with the FN tunneling model, linear curve fitting method has been normally utilized [8,20,41]. Nevertheless, data transformation is needed in this method owing to the limited models that can be presented in linear forms. Hence, nonlinear curve fitting method is employed using Datafit version 9.0.59 to fit the acquired *J-E* results in this work with the FN tunneling model. It is believed that the extracted results using nonlinear curve fitting method is more accurate due to the utilization of actual data and the minimization of data transformation steps required in the linear curve fitting [42,43]. Figure 7 shows the *J-E* results for the samples annealed in O_2_, Ar, and FG ambient, which fitted well with FN tunneling model. The extracted Φ_B_ values of these samples are presented in the Figure 4. The highest Φ_B_ value attained by the sample annealed in O_2_ ambient (3.72 eV) was higher than that of metal-organic decomposed CeO_2_ (1.13 eV) spin-coated on n-type GaN substrate [20]. No Φ_B_ value has been extracted for the sample annealed in N_2_ ambient due to the low *E*_B_ and high *J* of this sample, wherein the gate oxide breaks down prior to the FN tunneling mechanism.

Table 1 compares the computed Δ*E*_c_ values from the XPS characterization with the Φ_B_ value extracted from the FN tunneling model. From this table, it is distinguished that the *E*_B_ of the sample annealed in O_2_ ambient is dominated by the breakdown of IL as the obtained value of Φ_B_ from the FN tunneling model is comparable with the value of Δ*E*_c_(IL/GaN) computed from the XPS measurement. For samples annealed in Ar and FG ambient, the acquisition of Φ_B_ value that is comparable to the Δ*E*_c_(Y_2_O_3_/GaN) indicates that the *E*_B_ of these samples is actually dominated by the breakdown of bulk Y_2_O_3_. Since the leakage current of the sample annealed in N_2_ ambient is not governed by FN tunneling mechanism, a conclusion in determining whether the *E*_B_ of this sample is dominated by the breakdown of IL, Y_2_O_3_, or a combination of both cannot be deduced. Based on the obtained values of Δ*E*_c_(Y_2_O_3_/GaN), Δ*E*_c_(IL/GaN), and Δ*E*_c_(Y_2_O_3_/IL), the *E*_B_ of this sample is unlikely to be dominated by IL due to the acquisition of a negative Δ*E*_c_(IL/GaN) value for this sample. Thus, the *E*_B_ of this sample is most plausible to be dominated by either Y_2_O_3_ or a combination of Y_2_O_3_ and IL. However, the attainment of Δ*E*_c_(Y_2_O_3_/IL) value which is larger than that of Δ*E*_c_(Y_2_O_3_/GaN) value obtained for the samples annealed in Ar and FG ambient eliminates the latter possibility. The reason behind it is if the *E*_B_ of the sample annealed in N_2_ ambient is dominated by the combination of Y_2_O_3_ and IL, this sample should be able to sustain a higher *E*_B_ and a lower *J* than the samples annealed in Ar and FG ambient. Therefore, the *E*_B_ of the sample annealed in N_2_ ambient is most likely dominated by the breakdown of bulk Y_2_O_3_.

**Table 1:** **Comparison of the obtained Δ*E***_**c**_**and Φ**_**B**_**values**

	**XPS: conduction band offset**			***J*-*E***
	**Y**_**2**_**O**_**3**_**/GaN**	**IL/GaN**	**Y**_**2**_**O**_**3**_**/IL**	**Barrier height**
O_2_	3.00	3.77	0.77	3.72
Ar	1.55	1.40	0.15	1.58
FG	0.99	0.68	0.31	0.92
N_2_	0.70	−2.03	2.73	^a^

^a^Not influenced by FN tunneling. Therefore, barrier height is not extracted from the FN tunneling model.

## Conclusions

In conclusion, three different energy band alignment models of Y_2_O_3_/interfacial layer/GaN structure subjected to post-deposition annealing at 400°C in different ambients (O_2_, Ar, forming gas (95% N_2_ + 5% H_2_), and N_2_) have been established using X-ray photoelectron spectroscopy. It was proven that the dielectric breakdown field (*E*_B_) of the sample annealed in O_2_ ambient was dominated by the breakdown of IL, while the *E*_B_ of the samples annealed in Ar, FG, and N_2_ ambient was dominated by the breakdown of bulk Y_2_O_3_. The sample annealed in O_2_ ambient demonstrated the best leakage current density-breakdown field due to the attainment of the largest bandgap, the largest conduction band offset, and the highest barrier height value.

## Competing interests

The authors declare that they have no competing interests.

## Authors’ contributions

HJQ carried out all of the experimental work, data analysis of the obtained experimental results, and drafting of the manuscript. KYC had played a vital role in assisting HJQ in the experimental work and data analysis as well as in revising and approving the submission of the final manuscript for publication. Both authors read and approved the final manuscript.

## Authors’ information

HJQ received his MSc degree in 2010 from Universiti Sains Malaysia, Penang, Malaysia, where he is currently working on a PhD degree in Materials Engineering in the School of Materials and Mineral Resources Engineering. KYC received his PhD degree from the School of Microelectronic Engineering, Griffith University, Brisbane, Australia, in 2004. He is currently an associate professor with Universiti Sains Malaysia, Penang, Malaysia.
